# Dexmedetomidine Combined With Patient-Controlled Analgesia for Palliative Sedation in Terminal-Stage Cancer Patients With Refractory Pain: A Retrospective Analysis of Nine Cases

**DOI:** 10.1155/2024/4707707

**Published:** 2024-10-22

**Authors:** Na Li, Yumei Wang, Meng Cui

**Affiliations:** Department of Hospice Care, Shengjing Hospital of China Medical University, Shenyang 110022, China

**Keywords:** dexmedetomidine, palliative sedation, patient-controlled analgesia, refractory cancer pain, terminal-stage cancer

## Abstract

**Background:** Cancer-related pain is a pervasive symptom affecting the quality of life in patients with malignant tumors. For those with refractory pain, palliative sedation combined with pain management is recommended. Dexmedetomidine (DEX), known for its unique “awake sedation” effect, remains relatively unexplored when used in conjunction with patient-controlled analgesia (PCA) for terminal-stage cancer patients. This study aimed to assess the safety and efficacy of DEX for palliative sedation with PCA in patients experiencing refractory pain.

**Methods:** A retrospective analysis was conducted on terminal-stage cancer patients who received DEX for palliative sedation combined with PCA in a hospice ward between January 2020 and June 2023. Data collection included general patient information, laboratory tests, rating scales, pain and analgesia conditions, sedation details, palliative sedative effects, and changes in vital signs before and after sedation.

**Results:** Nine patients with terminal-stage cancer received DEX palliative sedation at doses ranging from 0.2 to 1.0 *μ*g/kg·h combined with PCA for refractory pain. After 1 h of sedation and at the maximum sedation dose, the Richmond Agitation-Sedation Scale scores significantly decreased (all *p* < 0.001). While heart rate, blood oxygen saturation, and respiratory rate remained stable, systolic blood pressure and diastolic blood pressure after 1 h of sedation were significantly lower than presedation levels (*p* = 0.040 and *p* = 0.044, respectively).

**Conclusion:** DEX emerges as a promising option for palliative sedation in terminal-stage cancer patients. When used in conjunction with PCA, DEX has been shown to effectively, safely, and stably control refractory pain without inducing adverse effects such as respiratory/circulatory depression.

## 1. Introduction

The global incidence of malignant tumors has exhibited an upward trend in recent years, posing a substantial burden on both families and society. Pain emerges as a predominant symptom associated with malignant tumors, with approximately 50% of patients reporting cancer-related pain, significantly impacting daily functioning and overall quality of life [1]. While standardized analgesic treatments effectively alleviate the majority of cancer-related pain, a notable 10%–30% of patients continue to experience refractory pain [2]. Patient-controlled analgesia (PCA) has demonstrated efficacy in enhancing analgesic outcomes for patients with refractory cancer pain attributed to its rapid onset, precision, safety, and individualized approach to pain management [3]. However, for terminal-stage cancer patients with only weeks or days remaining, severe psychological disturbances and symptoms of depression and anxiety can significantly impede the effectiveness of analgesic treatments. Current guidelines from the National Comprehensive Cancer Network (NCCN) for palliative care recommend considering palliative sedation for patients with severe refractory pain nearing the end of life, integrating pain management with reduced consciousness [4]. Dexmedetomidine (DEX) presents a promising option, possessing sedative, anti-anxiety, and analgesic properties while avoiding the serious adverse effects such as respiratory/circulatory depression that may be induced by midazolam (MDZ). The potential utility of DEX in palliative sedation for terminal-stage cancer pain remains an area of limited research and clinical experience.

This study provides a comprehensive examination of nine cases involving terminal refractory cancer pain treated with a combination of PCA and DEX palliative sedation. The safety and efficacy of DEX in this specific patient population were thoroughly analyzed and summarized, aiming to present an additional treatment option that merges palliative sedation with analgesia for patients with terminal refractory cancer pain.

## 2. Methods

### 2.1. Patients

The objective of this study was to conduct a retrospective analysis of terminal-stage cancer patients admitted the Hospice Ward of Shengjing Hospital of China Medical University between January 2020 and June 2023. The inclusion criteria for this study were as follows: (1) age not less than 18 years old; (2) patients must have been diagnosed by oncologists and the hospice physicians as being in the terminal-stage of malignant tumors; (3) patients must have exhibited refractory pain symptoms, receiving PCA and intravenous or subcutaneous DEX palliative sedation treatment prior to death; (4) patients must have refused invasive cardiopulmonary resuscitation measures such as endotracheal intubation, cardiac compression, and mechanical ventilation prior to death; and (5) patients must have died during hospitalization. The exclusion criteria for this study were as follows: Patients for whom detailed sedation, vital signs, or laboratory results could not be obtained were excluded from the study.

### 2.2. Methods

In this study, two hospice physicians systematically extracted data and collected patients' general information, including age, gender, tumor type, metastasis, tumor treatment, and an array of laboratory tests including white blood cells, hemoglobin, platelets, albumin, transaminase, total bilirubin, creatine kinase MB isoenzyme, creatinine, prothrombin time, activated partial thromboplastin time, fibrinogen content, D-dimer, fasting blood glucose, potassium, sodium, chloride, and calcium ions. Various rating scales were employed, encompassing the comprehensive cancer pain assessment, Quality of Life (QOL), Karnofsky Performance Status (KPS) [5], Palliative Performance Scale (PPS) [6], Nutritional Risk Screening 2002 (NRS2002) [7], and Richmond Agitation-Sedation Scale (RASS) [8]. The comprehensive cancer pain assessment involved the Numeric Rating Scale (NRS), which assessed pain intensity, type and quality of pain, location of pain, duration, factors exacerbating or relieving pain, and the current pain management plan. The QOL was conducted to comprehensively evaluate patients' overall well-being, encompassing their physical, psychological, social, and environmental aspects, along with satisfaction levels. Both the KPS and PPS were employed to assess patients' mobility and functional status. The NRS2002 tool was utilized to evaluate malnutrition in patients and to predict clinical outcome. The RASS was used to assess the quality and depth of palliative sedation, offering objective measures to gauge sedation levels effectively. Additionally, diverse palliative sedation-related data were gathered including duration of sedation, initial dose, and maximum dose. The RASS was employed to evaluate the quality and depth of palliative sedation. Vital signs, including blood pressure, heart rate, respiratory rate, and blood oxygen saturation, were documented before sedation, after 1 h of sedation, and at the maximum sedative dose drawing from the patient's medical record. This study received approval from the Ethics Committee of Shengjing Hospital affiliated to China Medical University (2022PS738K) and was conducted in strict accordance with the Helsinki Declaration, ensuring the complete protection of patient privacy.

### 2.3. Statistical Analysis

Statistical data analysis was performed using SPSS 25.0 (IBM Corp., Armonk, NY, USA), and a significance level of *p* < 0.05 was deemed statistically significant. Results were presented as the mean ± standard deviation for normally distributed measurement data, whereas for non-normally distributed data, the median and the 25th and 75th percentiles were used. Paired *t*-test was used to analyze the differences in variables before and after palliative sedation.

## 3. Results

### 3.1. Investigation of Terminal Cancer Pain

Between January 2020 and June 2023, a total of 418 patients who experienced terminal cancer pain were admitted to the hospice ward for palliative analgesic treatment. Among them, 69 individuals (16.5%) presented with refractory cancer pain. The effectiveness of analgesia for refractory cancer pain significantly improved through various interventions, including dosage escalation of opioids, management of adverse effects, opioid rotation, incorporation of additional adjuvant analgesics, interventional treatments, and alterations in the administration route. Despite these efforts, a subset of patients continued to experience inadequate pain relief, necessitating the implementation of terminal palliative sedation.

### 3.2. Analysis of General Data

This study included a total of 9 patients who underwent PCA and DEX palliative sedation. The median age of these patients was 67 years (range 56–80), comprising 4 males and 5 females. The patients presented with diverse primary malignancies: two cases of lung cancer, two cases of pancreatic cancer, and one case each of liver cancer, rectal cancer, gallbladder cancer, endometrial cancer, and ureteral cancer. All individuals exhibited multiple distant metastases and had undergone various active antitumor treatments before admission, including surgery, chemotherapy, radiotherapy, targeted therapy, and immunotherapy. At admission, eight patients experienced severe mixed pain, while one had severe visceral pain. Among these cases, seven manifested neuropathic pain. Assessment using the QOL, KPS, PPS, and NRS2002 indicated poor levels in quality of life, functional status, nutritional status, and a very short estimated survival time (Table 1). Laboratory test results revealed prevalent infections, anemia, hypoalbuminemia, abnormal liver and kidney function, coagulation dysfunction, and ion disorders among most patients (Table 2).

### 3.3. Analysis of PCA and Palliative Sedation

After admission, a revised pain management protocol was implemented for all patients, involving the escalation of opioid dosages, symptomatic treatment for adverse effects, opioid rotation, and the addition of adjuvant analgesics. Despite these interventions, pain persisted without relief in nine patients. Consequently, these individuals underwent PCA, with three receiving hydromorphone injections and six receiving morphine injections through subcutaneous or intravenous PCA. The detailed pain management protocol is outlined in Table 3. While there was some improvement in pain relief compared to previous treatments, the patients expressed ongoing dissatisfaction despite repeated increases in opioid dosages.

Concurrently, psychological symptoms such as anxiety, agitation, and insomnia were evident in the patients. Both the patients and their families strongly advocated for palliative sedation as a means of reducing consciousness and alleviating pain. Following evaluation by two attending physicians and obtaining informed consent from the patients and their families, a continuous infusion of DEX was administered, starting at an initial dose of 0.2 *μ*g/kg·h and gradually increasing based on the patient's condition, up to a maximum dose of 1.0 *μ*g/kg·h. Throughout palliative sedation, the patients maintained a level of consciousness ranging from drowsiness and lethargy, yet they remained arousable. There was a notable improvement in psychological well-being, and pain levels became tolerable. Ultimately, the patient passed away with dignity, calmness, and peace.

### 3.4. Analysis of Vital Signs Before and After Sedation

Vital sign data, including systolic blood pressure, diastolic blood pressure, heart rate, respiratory rate, and blood oxygen saturation, were meticulously documented before and after sedation (Table 4). Following 1 h of sedation and at the maximum sedation dose, the RASS scores exhibited a significant decrease compared to the presedation values (all *p* < 0.001). While there was a decrease in heart rate and respiratory rate after sedation, these changes were not statistically significant compared to the presedation values. Additionally, blood oxygen saturation did not show a significant alteration after sedation, despite a downward trend. Systolic and diastolic blood pressure, on the other hand, exhibited a significant decrease after 1 h of sedation (*p*=0.040 and *p*=0.044, respectively), although no significant change was observed at the maximum sedation dose.

## 4. Discussion

This retrospective study, conducted at a single center, offers valuable insights into the practice model and characteristics of palliative sedation for managing refractory pain in terminal-stage cancer patients. The initial DEX dose for all patients in this study was set at 0.2 *μ*g/kg·h, with a maximum dose of 0.5–1.0 *μ*g/kg·h, which is slightly higher than the 0.3–0.5 *μ*g/kg·h used in a previous study on palliative sedation [9] but still falls within the Food and Drug Administration (FDA)–recommended dosing range of 0.2–1.0 *μ*g/kg·h [10]. Contrary to the FDA recommendation of a loading dose of 1 *μ*g/kg administered 10 min prior to procedural sedation, studies on elderly patients have cautioned against this practice. Administering 0.5 or 1.0 *μ*g/kg DEX within 10 min for sedation induction was found to result in excessive sedation in 60% of patients in one study [11]. Additionally, another study indicated that a loading dose ≥ 0.7 *μ*g/kg increased the incidence of cardiovascular side effects such as arterial hypertension [12]. Considering the uncertain safety profile and the absence of an urgent need for rapid sedation in these patients, our study refrained from administering a loading dose.

The vital signs of 9 patients were monitored, revealing a notable but transient decrease in blood pressure 1 h after sedation (*p*=0.040 for systolic BP and *p*=0.044 for diastolic BP). However, other vital indicators such as heart rate, respiratory rate, and blood oxygen saturation did not exhibit statistically significant changes. No serious adverse effects, such as respiratory/circulatory depression, were observed during palliative sedation, and other side effects like delirium, agitation, dystonia, and tremors were also absent. It is noteworthy that a review of DEX has cautioned about the potential risks of adverse effects, including arterial hypertension, hypotension, bradycardia, QT prolongation, and atrioventricular block [13]. The hemodynamic effects, such as transient hypertension, bradycardia, and hypotension, arise from the drug's peripheral vasoconstrictive and sympatholytic properties [14]. Variability in vital signs during palliative sedation with DEX is noted across studies. Our earlier research on end-stage dyspnea showed transient hypotension and reduced respiratory rates after sedation, potentially attributed to variances in patient populations, with a predominant focus on dyspnea patients, wherein respiratory rates typically increased initially and decreased following dyspnea control [15]. In contrast, a study on advanced heart failure patients reported no significant vital sign changes [16]. Furthermore, in another study, 17 of 20 patients experienced hypotension, leading to DEX discontinuation in two cases [17]. These discrepancies may stem from variations in patient characteristics or monitoring methods and timing of vital signs, necessitating further research with larger sample sizes and stricter monitoring protocols to validate these findings.

Palliative sedation is defined as the application of appropriate sedative drugs to lower a patient's level of consciousness, aiming to alleviate refractory or intolerable painful symptoms in terminal-stage patients and enabling them to experience reduced or no pain at the end of life [4, 18]. A systematic review has identified delirium (41%–83%), pain (25%–65%), dyspnea (16%–59%), and mental and psychological symptoms (16%–59%) as the primary refractory symptoms necessitating palliative sedation [19]. In this study, a comprehensive assessment was conducted upon the admission of nine patients to confirm their terminal stage of malignant tumors and severe physical and mental pain, meeting the indications for palliative sedation. The combination of PCA and DEX effectively relieved refractory pain, easing patients' suffering, anxiety, and other negative emotions. This approach greatly enhanced both the quality of life and the dying process during end-of-life care, while also providing comfort to grieving family members and ultimately promoting a peaceful passing.

MDZ is the most commonly employed palliative sedative among cancer patients, providing deep sedative effects but also posing risks of respiratory/circulatory depression [20–23]. Considering that deep sedation is not always necessary, DEX emerges as an alternative. DEX, used in perioperative and intensive care settings, binds to central nervous system alpha-2 adrenergic receptors, inducing a state akin to non-rapid eye movement sleep and providing sedative, anti-anxiety, and analgesic effects [9, 24]. In this study, the majority of patients remained in a state of mild sedation after DEX sedation, with a significant decrease in RASS scores, signifying reduced consciousness levels. Effective pain control is achieved, in part, due to the analgesic properties of DEX, which are derived from various mechanisms demonstrated in previous studies, including supraspinal, ganglionic, spinal, and peripheral effects of alpha-2 adrenergic agonists [25, 26]. Additionally, DEX can lower a patient's consciousness level, thereby diminishing their perception and sensitivity to pain. In contrast to MDZ, DEX provides the unique advantage of “awake sedation,” making it a viable option for patients not requiring deep sedation.

In discussions surrounding palliative care, it is crucial to address various ethical considerations, including the criteria for determining which patients may be appropriate candidates for palliative sedation and the informed consent process for such interventions. When palliative sedation is deemed necessary, a multidisciplinary consultation and assessments by at least two physicians are required to confirm that the patient is in the terminal stage of a malignant tumor and exhibits refractory symptoms that are not manageable by standard palliative care. A thorough discussion must then take place with the patient and/or their family regarding the patient's condition, prognosis, sedation objectives, methods of administration, and potential adverse effects. Informed consent for sedation must be obtained from the patient and/or their family, and the informed consent should detail potential adverse reactions, including inadequate sedation, excessive sedation that may suppress respiration or impair expectoration, psychiatric abnormalities, and severe reactions that could threaten the patient's life. Following this, an appropriate sedation plan should be carefully selected and implemented, with continuous monitoring of the patient's symptoms and signs. Sedatives should be adjusted to meet the patient's individual needs.

A frequently raised concern is whether palliative sedation might shorten survival time. In this study, the duration of sedation varied from a minimum of 2 days to a maximum of 38 days. A previous systematic review indicated that the average or median duration of palliative sedation ranged from 0.8 days to 12.6 days [27]. Another study compared survival between sedated and nonsedated patients, revealing no notable disparities in survival outcomes between the two groups [28]. It is crucial to note that palliative sedation is a short-term intervention intended to alleviate refractory symptoms in terminal patients. It is distinct from euthanasia and does not shorten life.

In conclusion, DEX has emerged as a promising option for palliative sedation in terminal-stage cancer patients. Its use in combination with PCA has shown to be effective, safe, and stable in controlling refractory pain, without causing adverse effects such as respiratory/circulatory depression. Especially for patients requiring “awake sedation” to manage refractory pain, DEX holds significant potential to improve their quality of life. The clinical application and promotion of DEX in these settings are strongly recommended.

However, as this study is retrospective in nature and primarily focused on terminal-stage cancer patients, its findings are inherently limited. Further prospective research and clinical trials are needed to thoroughly assess DEX's efficacy in managing a broader range of refractory symptoms in terminal patients. Continued investigation and expanded clinical insights will be crucial in refining DEX's role in palliative care, ultimately enhancing both comfort and quality of life for those nearing the end of life.

## Figures and Tables

**Table 1:** General patient information.

	Case 1	Case 2	Case 3	Case 4	Case 5	Case 6	Case 7	Case 8	Case 9
Age	66	62	80	69	70	67	64	56	67
Gender	F	F	F	F	M	F	M	M	M
Primary tumor	Pancreas	Ureter	Liver	Lung	Rectum	Endometrium	Pancreas	Lung	Gallbladder
Metastasis	Liver, retroperitoneum	Bladder, retroperitoneal	Bone	Pleura, bone	Bone, lung, liver, pleura, iliopsoas	Peritoneum	Liver	Bone	Bone, lung, liver, pancreas, peritoneum
Previous treatment									
Surgery	No	Yes	No	No	Yes	Yes	No	Yes	Yes
Chemotherapy	Yes	No	Yes	Yes	Yes	Yes	Yes	Yes	Yes
Radiotherapy	Yes	No	No	No	No	No	Yes	No	Yes
Others	No	Yes	Yes	Yes	Yes	No	No	Yes	Yes
QOL	19	21	18	25	20	28	22	27	24
KPS	10	20	20	20	20	20	10	20	20
PPS	10	30	20	20	20	20	20	30	20
NRS2002	4	4	5	4	5	5	5	4	4
NRS	8	9	9	10	9	8	9	10	9
Location of pain	Upper abdomen, lower back	Lower abdomen	Upper abdomen, left ribs	Back, left upper limb	Lower abdomen, perineum	Abdomen	Upper abdomen, lower back	Lower back, lower limbs	Abdomen, lower back
Quality of pain	Distending, numb	Dull, scalding	Distending, prickling	Dull, numb	Cutting, burning	Distending	Distending, burning	Prickling, shooting	Dull, bursting
Duration of pain	24 h	24 h	24 h	24 h	24 h	24 h	24 h	24 h	24 h
Type of pain	Visceral, neuropathy	Visceral, neuropathy	Somatic, visceral	Somatic, visceral, neuropathy	Visceral, neuropathy	Visceral	Visceral, neuropathy	Somatic, neuropathy	Visceral, neuropathy

Abbreviations: KPS = Karnofsky Performance Status, NRS2002 = Nutritional Risk Screening 2002, NRS = Numerical Rating Scale, PPS = Palliative Performance Scale, and QOL = Quality of Life.

**Table 2:** Laboratory test results of patients.

	Case 1	Case 2	Case 3	Case 4	Case 5	Case 6	Case 7	Case 8	Case 9	Reference value
WBC (×10^9^/L)	26.7	11.9	3.8	28.9	13.6	7.7	9.9	10.4	8.2	3.5–9.5
HGB (g/L)	86	109	90	112	111	115	57	120	90	Female: 110–150; male: 130–172
PLT (×10^9^/L)	88	342	100	302	286	218	25	209	226	135–350
ALB (g/L)	20.6	40.5	22.9	25.4	31.1	28.7	24.2	36.5	22.8	35–53
ALT (U/L)	24	16	58	3	14	19	14	34	39	0–40
AST (U/L)	93	15	62	8	41	26	45	15	12	5–34
TBIL (*μ*mol/L)	64.1	13.2	24	7.1	32	28.5	35.7	10.4	20.9	3.4–20.5
CK-MB (U/L)	53	10	8.3	15.1	110.1	6.3	9.4	27.6	17.6	0–24
CREA (*μ*mol/L)	92	85.7	106	57.3	89.8	66	96	60.1	33.8	Female: 45–84; male: 59–104
PT (S)	24.8	12.5	12.6	14	13	11.3	15.9	12.1	15	9.4–12.5
APTT (S)	40.7	25.1	31.4	27.6	32	28.1	31.8	25.7	33.8	21–37
FIB (g/L)	1	4.72	2.58	3.8	2.99	1.5	2.93	3.8	4.02	2–4
DD (*μ*g/L)	4538	365	3283	562	2251	708	1458	375	1296	0–252
FPG (mmol/L)	4.21	8.74	5.57	6.24	5.65	5	8.18	5.28	4.26	3.9–6.11
K+ (mmol/L)	3.98	4.13	4.21	3.65	4.32	4.5	3.8	3.65	3.99	3.5–5.5
Na+ (mmol/L)	141.6	121.7	134.1	126.4	133.4	145.8	131	138.2	136.7	136–145
Cl− (mmol/L)	103.2	82.8	105.9	89.9	94.9	103.1	93.4	102.2	99.5	96–108
CA (mmol/L)	1.89	2.24	2.05	1.99	2.26	2.47	1.71	2.01	1.83	2.1–2.55

Abbreviations: ALB = albumin, ALT = alanine transaminase, AST = aspartate transaminase, APTT = activated partial thrombin time, CA = serum calcium, CK-MB = creatine kinase isoenzyme, Cl− = serum chloride, CREA = creatinine, DD = D-dimer, FPG = fasting plasma glucose, FIB = fibrinogen content, HGB = hemoglobin, K+ = serum potassium, Na+ = serum sodium, PLT = platelet, PT = prothrombin time, TBIL = total bilirubin, TT = thrombin time, and WBC = white blood cell.

**Table 3:** Pain management plan for patients before admission, after admission, and before sedation.

	Pain management before admission	Pain management after admission	Pain management before sedation
Case 1	Oxycodone SR 80 mg q12 h Morphine IR 30 mg as needed	IV hydromorphone 1.5 mg/h bolus of 3 mg	IV hydromorphone 2.5 mg/h bolus of 5 mg

Case 2	Oxycodone SR 60 mg q12 h Morphine IR 30 mg as needed	Oxycodone SR 120–200 mg q12 h Morphine IR 60–120 mg as needed ⟶IV morphine 15 mg/h bolus of 30 mg	IV morphine 30 mg/h bolus of 60 mg

Case 3	Oxycodone SR 30 mg q12 h Morphine IR 20 mg as needed	IV hydromorphone 0.25 mg/h bolus of 0.5 mg	IV hydromorphone 0.5 mg/h bolus of 1 mg

Case 4	Oxycodone SR 200 mg q12 h Morphine IR 120 mg as needed	IV morphine 15 mg/h bolus of 30 mg	IV morphine 40 mg/h bolus of 80 mg

Case 5	Transdermal fentanyl 100 *μ*g/h q72 h Morphine IR 30 mg as needed	Transdermal fentanyl 150–200 *μ*g/h q72 h Morphine IR 40–60 mg as needed ⟶IV morphine 5 mg/h bolus of 10 mg	IV morphine 8 mg/h bolus of 16 mg

Case 6	Transdermal fentanyl 100 *μ*g/h q72 h Morphine IR 30 mg as needed	Transdermal fentanyl 150 *μ*g/h q72 h Morphine IR 40 mg as needed ⟶IV morphine 5 mg/h bolus of 10 mg	IV morphine 40 mg/h bolus of 60 mg

Case 7	Transdermal fentanyl 50 *μ*g/h q72 h Morphine IR 20 mg as needed	Transdermal fentanyl 100 *μ*g/h q72 h Morphine IR 30 mg as needed ⟶IV hydromorphone 1.5 mg/h bolus of 3 mg	IV hydromorphone 2 mg/h bolus of 4 mg

Case 8	Transdermal fentanyl 250 *μ*g/h q72 h Morphine IR 60 mg as needed	IV morphine 10 mg/h bolus of 20 mg	IV morphine 20 mg/h bolus of 40 mg

Case 9	Oxycodone SR 90 mg q12 h Morphine IR 60 mg as needed	IV morphine 5 mg/h bolus of 10 mg	IV morphine 25 mg/h bolus of 50 mg

Abbreviations: IR = immediate release and SR = sustained release.

**Table 4:** Patients' palliative sedation details and changes in vital signs.

	Case 1	Case 2	Case 3	Case 4	Case 5	Case 6	Case 7	Case 8	Case 9		*p* value
Initial dose (*μ*g/kg·h)	0.2	0.2	0.2	0.2	0.2	0.2	0.2	0.2	0.2	—	
Maximum dose (*μ*g/kg·h)	0.6	0.7	0.6	0.9	0.8	0.5	0.5	1.0	0.7	0.7 ± 0.2	
Duration of sedation (day)	2	38	2	22	5	6	6	4	6	6 (3, 14)	
Before sedation											
RASS	2	2	2	1	3	2	3	3	2	2 (2, 3)	
Systolic BP (mmHg)	97	94	98	128	103	95	93	92	91	99 ± 11	
Diastolic BP (mmHg)	63	60	58	68	77	68	58	53	54	63 ± 7	
HR (bpm)	100	118	92	110	100	80	110	96	92	100 ± 12	
RR (bpm)	24	20	20	19	26	20	20	22	24	22 ± 2	
SpO2 (%)	95	98	96	98	96	98	97	98	97	97 ± 1	
1 h after sedation											
RASS	1	1	0	0	1	1	1	2	0	1 (0, 1)	< 0.001
Systolic BP (mmHg)	90	76	105	112	107	72	86	82	88	91 ± 14	0.040
Diastolic BP (mmHg)	58	50	62	56	78	50	54	52	60	58 ± 9	0.044
HR (bpm)	98	110	96	116	94	86	106	98	90	99 ± 10	0.803
RR (bpm)	20	20	18	21	24	18	21	20	18	20 ± 2	0.076
SpO2 (%)	94	97	95	99	97	98	96	96	98	97 ± 2	0.397
At maximum sedative dose											
RASS	−2	0	−1	−1	0	−2	−2	0	−1	−2 (−1, 0)	< 0.001
Systolic BP (mmHg)	110	96	86	105	95	84	90	95	95	95 ± 8	0.310
Diastolic BP (mmHg)	64	58	58	59	68	58	61	55	60	60 ± 4	0.105
HR (bpm)	92	96	88	110	82	86	120	88	88	94 ± 13	0.159
RR (bpm)	23	18	15	21	25	16	22	16	18	19 ± 4	0.055
SpO2 (%)	96	98	90	98	97	99	98	92	94	96 ± 3	0.255

*Note:* The data are presented as mean ± standard deviation (SD) or median (interquartile range, IQR). Paired *t*-tests were utilized to analyze the differences in blood pressure, heart rate, respiratory rate, and blood oxygen saturation 1 h after sedation versus before sedation, as well as the maximum sedative dose versus before sedation, respectively.

Abbreviations: BP = blood pressure, bpm = beats per min, HR = heart rate, RASS = Richmond Agitation-Sedation Scale, RR = respiratory rate, and SpO2 = oxygen saturation.

## Data Availability

The data that support the findings of this study are available from the corresponding author upon reasonable request.
